# On a Remedy (the Ambrosia Trifida) for Mercurial Salivation

**Published:** 1846-12

**Authors:** Wm. Robertson

**Affiliations:** Harrodsburgh, Ky.


7. *On a Remedy (the Ambrosia Trifida) for Mercurial Salivation.*—By Wm. Robertson, M.D., of Harrodsburgh, Ky.

One of the most common plants on our farms, possesses, as I have discovered, more prompt and efficacious remedial powers, in the cure of mercurial salivation, than any article I have ever seen tried for that loathsome disease. During a practice of forty years, I have seen the disease in all its forms, and various remedies employed for it, but do not recollect to have ever witnessed an obvious curative influence exercised by any of them.

The remedy I have lately adopted, in every case in which I have tried it, has proved a speedy and effective cure, relieving the patients in from six to eight hours, removing every symptom of salivation. However, I would observe, that all these cases have been of a mild character, or in the incipient stages. What influence this remedy would exert in those violent cases of the disease occasionally met with in practice, attended with extensive swelling, ulceration, sloughing, and falling out of the teeth, I am unable to say, having met with no such case since my adoption of the article; but I think it probable that such a case would call for the use of other remedies. Nevertheless, the use of this remedy, in the commencement of such cases, would, very probably, arrest their progress, and prevent their attaining an aggravated form. In this view, I am sustained by the result of a case that came under my notice within the last month. In this case, the power and influence of this medicine, to control mercurial salivation, were most strikingly exemplified. It was that of a female, aged thirty-five, in the eighth month of her pregnancy, of delicate frame and phlegmatic temperament, and predisposed to hysteria. She was advised, for habitual costiveness and torpid liver, to take one or two doses of calomel, milder purgatives having procured only momentary relief. The calomel was retained about thirty hours, although followed by a large dose of castor oil, in ten or twelve hours. The consequence was, a violent attack of mercurial salivation. Within twenty-four hours from the attack, some unusual symptoms having manifested themselves, the family became alarmed, and I was hastily called to visit her, five miles in the country.

The bowels having been evacuated by injections, I found the patient without fever, and only complaining of the salivation. The gums and mucous membrane of the mouth were inflamed, a little swelled, and had a soft, puffy appearance; the whole surface was covered with thick viscid mucus, adhering with unusual firmness, and so offensive in smell and taste to the patient, that every effort to discharge it was attended with nausea and vomiting; a putrid effluvium was exhaled with every breath, along with the mercurial fetor, perceptible and offensive to the bystanders. It was this symptom that had alarmed the family: they concluded that mortification had already taken place. All perception of taste had ceased, and food and drinks were rejected with disgust. The putrid smell perceptible in the breath evidently proceeded from the viscid mucus, adhering to the mouth and throat, acquiring a putrescent tendency, from being detained there long after the secretion was thrown out from the secreting glands, &c. This was proved by an examination of the secretion; when discharged (as it was with great effort) into some vessel, the same putrid smell was present, and the mucus was about the consistency of the white of an egg.

This case of pure mercurial salivation—I say pure, because this disease is very generally accompanied by other diseased conditions of the system—afforded me the best opportunity I had seen of testing the powers of the remedy. I immediately procured from an adjoining field a large handful of the green leaves; poured on them in a suitable vessel one quart of boiling water; as soon as it was cooled sufficiently, the patient was directed to wash the mouth and throat freely every half hour; nothing else was used except the common soda powders; they were given every three hours, in an effervescing state.

I remained with the patient six hours. By that time the mouth and throat were cleared of the thick viscid mucus; the nausea and vomiting had ceased entirely; the natural taste was nearly restored; the patient felt greatly relieved, and partook of some light food with relish. The next day she was still improving and comfortable, and on the third day, within forty-eight hours from the time of commencing the use of the remedy, every symptom of salivation was removed, and the female was engaged in her usual domestic vocations.

I will give another case, which occurred within the last two weeks, because there is a fact connected with it, giving rise to an opinion that the remedy may prove beneficial to inflammation in mucous membranes, arising from other causes than mercury. A gentleman, from bathing in a river, took cold. He called on me, complaining of headache, sore throat, a stiff neck. He was bled; some active cathartic pills, containing a small quantity of calomel, were given, with directions to use them so as to keep the bowels in a solvent condition; to use a light diet, and apply vol. liniment to the throat. Three days afterwards he called on me, to inform me that the pills, as used, had not been active enough, and that he was salivated; the sore throat still continued without abatement.

I gave him a handful of the fresh leaves, and directed him how to use the infusion. He afterwards informed me that twenty-four hours' use of the remedy removed every symptom of salivation, and that the sore throat had also been cured. He further informed me, that at the time he received the remedy, he felt so badly about the mouth and throat, that he did not expect he would be able to preach for a week (he is a minister of the Gospel); but that, after using the remedy, he found himself as able to preach at the end of two days, as ever he had felt in his life.

May not this remedy prove beneficial as a local application in leucorrhœa, prolapsus uteri, and gonorrhœa, also in various affections of the throat? I shall certainly, in future, extend its use to diseases of this character; and I hope that practitioners of medicine, especially those residing in districts where the plant abounds, may be induced to give it a trial, and report to the profession the result of their practice.

This plant is known in all parts of Kentucky, and is known to all our farmers under the popular names of horseweed, richweed, horsemint, and horsecane; but it is an entirely different plant from that described in the appendix to the fourth edition of *Wood and Bach's Dispensatory*, at page 1137, under the title of *Collinsonia Canadensis*, and vulgarly known by names similar to those applied to the Kentucky plant.

I was induced to make trial of this plant in mercurial salivation, from the fact that this plant, when given to a horse affected with a disease called slabbering, effects a complete cure of the disease in a few hours.

This salivation, or slabbering disease in the horse, doubtless proceeds from some diseased condition of the salivary glands. About two years ago, passing a field where the plant was abundant, its effect on the salivated horse occurred to my mind, and immediately a question suggested itself—that if this remedy can exert such speedy and such surprising effects on the salivary glands of the horse, may it not possess properties that would render it useful and beneficial in salivation in the human subject? Under this impression, I resolved on a trial of its powers in the first case that should present itself. The trial convinced me that it possessed powers for relieving and curing mercurial salivation, greatly surpassing any means I had hitherto used; and subsequent experience has firmly established that conviction.

The effects produced by the local application of the infusion in the human subject, induces me to think that the effect it produces on the horse, does not arise from the plant taken into the stomach and reaching the diseased glands through the medium of the circulation, but that the direct application of the juice of the plant, while the horse is chewing it, effects the cure. It has so happened, that all the cases in which I have had occasion to use the remedy, have occurred during the spring, summer, or fall, when the plants are in a green state. I have the dried leaves, but have never used them; whether the leaves lose any of their virtues by drying, I am unable to say. I have never heard of the plant being used, in any shape, as a medicine, until I tried it as a remedy for salivation.

[Dr. Robertson was polite enough to send us, with the above communication, some dried specimens of the above plant, which we submitted to our friend, Dr. R. E. Griffith, an able botanist, from whom we have received the following note:—

Dr. Hays:—Dear Sir,—The plant you left with me appears to be *Ambrosia Trifida*, though, from the absence of flowers or fruit, it is difficult to decide with absolute certainty; at the same time, the character of the leaves and stem are so striking, as to leave little doubt on the subject.

Torrey and Gray (*Flor. Nor. Amer.*, ii. 290,) describe it as follows:—"Stem tall and stout, hairy, rough; leaves scabrous and hairy, deeply three-lobed; the lobes oval, lanceolate, acuminate serrate; the lower leaves often five-lobed; petioles narrowly winged, ciliate, racemes often paniculate; fruit (fertile involucre) turbinate-obovoid, with a short conical pointed apex, six ribbed, the ribs terminating in as many cristate tubercles.

"Low grounds, and along streams, Canada to Georgia, and west to Louisiana and Arkansas. Aug.—Sept. annual."

It is also noticed by Riddell (*Synop. Flor. West. States*, No. 1014) as everywhere abundant: he gives the vulgar name of bitter-weed to it. Rafinesque (*Med. Flor.*, ii. 190) speaks of it, and says that it is called horseweed, one of the names given by Dr. Robertson, and states that the species of ambrosia are antiseptic.

The *A. Trifida* has not, as far as I can ascertain, been employed as a remedial agent, though some of the other species have been used, with some success, as febrifuges. Should the present plant, on a more extended trial, be found to be as successful in cases of mercurial salivation as is shown by Dr. Robertson, it will be a very important addition to the *materia medica*. It is to be found in abundance in the vicinity of Philadelphia. It is probable that the *A. Elatior*, or rag-weed, so common in all our fields, would prove still more efficacious, as its sensible properties are much more developed than in the present plant. Yours, &c.,

R. E. Griffith, M.D.]

*Am. Jour. of Med. Science*, in *St. Louis Med. & Surg. Jour.*

—

Dr. John B. Beck, *on Emetics in Children.*—The name of the Brothers Beck has been rendered classical in American literature, by the great work upon Medical Jurisprudence, composed by them in company. Our own fellow citizen, Dr. John B. Beck, has long been known to the profession here and abroad, as the able professor of Materia Medica in the College of Physicians and Surgeons in this city; as a man of vigorous intellect, a scholar of extensive learning, a physician of great sagacity, and the author of many valuable papers indicative of sound judgment, and possessed of the highest practical value. His essay on Laryngitis (see his Medical Researches) as we will hereafter show, contains much that has been appropriated, without acknowledgement, by subsequent writers on this disease; and no abler argument has yet appeared upon the non-contagiousness of yellow fever, than that republished in the same volume. In the September number of our cotemporary, the N. Y. Journal of Medicine, Dr. B. has published as a sequent to a previous one on the use of opium in early age, a paper upon the "effects of emetics in the young subject," so characteristic of its author, so interesting, so practically true and important, that we shall make not the slightest apology, for laying before our readers very copious extracts.

"With the exception of cathartics, there is no class of remedies more generally resorted to in the management of the diseases of children, than emetics; and in a large number of cases, there is certainly none more useful. They are active agents, however, and like all agents of this description, are capable of doing good or evil, according to the manner in which they are given.

"In a previous paper, I endeavored to point out how the effects of opium were modified in the infant subject. On the present occasion I propose to pursue a similar investigation in relation to emetics.

"As it regards *the mere mechanical act of vomiting, young children perform it more easily than adults.* This is a fact which has long been observed by practical men, and about which there can be no question. It is, no doubt, a wise provision of the Creator, to enable the child to relieve itself from the effects of an overloaded stomach, to which it is so constantly liable in the early period of its existence. Although the fact has thus long been known, and the intention of it is obvious, yet the reasons have not been so well understood. They appear to be the two following.

"In the first place, from the experiments of Majendie, in relation to the manner in which vomiting is performed, it would seem that, in that process, the stomach is in a great measure passive, and that a certain degree of pressure upon it from the surrounding organs is absolutely necessary, before vomiting can be accomplished. This pressure is made by the contraction of the diaphragm from above, and of the abdominal muscles from below, upon the viscera surrounding the stomach. As a matter of course, the pressure thus exerted will be greater or less, according to the volume of the viscera. Now it is well known that in the early periods of life, the abdominal viscera have a much larger proportional size than they have in the adult. This is particularly the case with the liver. In early life, therefore, during the act of vomiting, the pressure made upon the stomach by the surrounding organs must necessarily be greater than it is in the adult, and in consequence of this, the greater the ease with which the organ is evacuated.

"In the second place, the shape of the stomach in the infant is more favorable to the easy evacuation of its contents. That the stomach undergoes successive changes in its shape, from birth onwards, is a fact, which although but recently investigated, is, I believe, well established, and for its elucidation we are indebted to the labors of Prof. Shultz, of Germany.

"The foregoing considerations would seem to account very satisfactorily, both physiologically and anatomically, for the fact with which we started, that the mechanical act of vomiting is performed with greater ease in the child than in the adult. If vomiting, then, be induced in a child by mild agents, the whole process is performed with greater facility than by the adult. This, then, is the *first* peculiarity in the effects of Emetics in children.

"If, on the other hand, *Emetics of an active and debilitating character, and which produce much nausea, be used, the effects are more uncertain and energetic than in the adult.* The articles to which I allude are the antimonial emetics, and these are frequently hazardous to young children, and that, too, when used in doses not peculiarly large. The immortal Sydenham seems to have been fully aware of this fact. In speaking of the continued fever of 1661, 2, 3, and 4, he says, 'it has often been a difficulty with me, when called to infants and children in a fever, and observing an emetic indicated, whereby they might have been preserved from danger, that I durst not give them this infusion *(crocus metallorum)*, for fear of a bad consequence.' * * * * * *

"I have known a case occuring in this city in which the thirtieth part of a grain of Tartar Emetic given to a child a year old, labouring under croup, produced such severe and protracted vomiting, together with general prostration, as to require stimulants to save life. Some years since I was called to see a child about three years old, who had been attacked with scarlet fever. The symptoms at first were mild, and no danger was apprehended in the case, when it was suddenly taken with such alarming symptoms of prostration as to call for a consultation. On inquiry, I found that the attending physician had been prescribing small doses of Tartar Emetic. Notwithstanding the use of stimulants, the child died in an hour or two after I saw it. I then suspected, and have since been confirmed in the correctness of the suspicion, that the medicine had no little agency in bringing about the fatal result. The child was naturally delicate, and there certainly was nothing in the symptoms of the case to account for such a termination.

"The foregoing facts would seem sufficient to show the *uncertainty* as well as *energy* with which Tartar Emetic operates on the young subject, and the causes are obvious. * *

"While Tartar Emetic operates in this way on the young subject, Ipecacuanha is never known to be followed by any injurious consequences. To the young infant it may be given, not only with impunity, but frequently with the greatest benefit. Why this is so must be manifest, if we reflect for a moment upon the peculiar properties of the two articles. Although both are emetics, yet they differ widely from each other in many important respects. The one is a mild article, limited in its operation to the stomach, upon which it never produces anything like local irritation, even when given in large doses. The other, besides acting as a powerful emetic, is a direct sedative capable of producing general prostration, and in some cases acting as a local irritant to the stomach and bowels, showing itself in excessive vomiting and diarrhœa. * *

"Now the due understanding of these peculiarities is evidently of the highest importance in the use of Emetics in children, and upon the mind of the student and young practitioner especially they cannot be too deeply impressed. From the manner in which medicines are treated of in classes, in most of the books of Materia Medica, and in the lectures on that subject, the student is insensibly led into the belief of a greater resemblance between them than really exists in nature, and it is only after he has had some experience of his own, that the errror is corrected. He cannot, therefore, too early in his career learn that all classifications are artificial—not founded in nature—that medicines are arranged in classes, merely for the sake of convenience; not because the articles under each class are precisely alike, but because they resemble each other in some one or more important feature, while in other respects they differ greatly. No two medicines, even in the same class, are precisely similar, and in acquiring a knowledge of them, the study of the points of difference is even more important than those in which they resemble each other.

"From the foregoing considerations, it appears to me that some inferences of practical value, to the young practitioner at least, may be deduced.

"1. As a general rule, we need not be afraid of vomiting the youngest child, provided the means used be mild—such as ipecacuanha, &c. The mere act of vomiting is attended with no danger, while the remedial agency of an emetic is one of great power and value.

"2. The vomiting induced by the preparations of antimony ought to be resorted to with great caution in very young children, and should never be used except in those cases where a sedative effect is required, and can be borne with safety.

"3. The *continued use* of Tartar Emetic in young subjects, cannot be too specially guarded against. It is in this way, probably, that it is so apt to prove injurious. A single dose, even though it vomits very freely, may be borne with comparative impunity, while its repetition may keep up nausea and intestinal irritation, so as to cause injurious prostration. This is very likely to happen in cases of a chronic character, like hooping-cough. Although mild emetics are among our best remedies in this disease—and where the subject is old enough, a single emetic of antimony is frequently exceedingly beneficial—yet the repeated use of antimonial emetics, as is too often the case, appears to me to be a great error in practice. It is not indicated by the nature of the symptoms, and violates a great rule which ought always to be observed in the management of chronic cases, and that is, not to break down unnecessarily the strength of the patient. Again, in ordinary catarrhal affections in children, a good deal of mischief is frequently done by the continued use of expectorant mixtures containing this active article. The Hive Syrup of Dr. Coxe, which is now in every family, and is given on the slightest occasion to infants, without even consulting a physician, has, I am convinced, done a great deal of harm. I say this without wishing to undervalue this preparation. In proper cases it is really a useful article, but persons out of the profession ought to know that its principal efficacy is owing to the quantity of Tartar Emetic which it contains, and that the indiscriminate use of it in cases where mild articles are required, must be injurious.

"4. As the effect of Tartar Emetic on the system cannot always be measured by its emetic operation, even in the adult, this fact ought to serve as a caution against the too common practice of giving repeated doses of it to produce vomiting in children, when they happen to be narcotized. While it fails to vomit, it may still operate as a poison to the system. In all cases of this kind, the proper method of treatment is, not to push the emetic, but to endeavor to restore the sensibility of the patient, and then sometimes vomiting comes on at once.

"5. In using Tartar Emetic in children, especial regard should be had to their constitutions. In those naturally delicate, and especially where the scrofulous diathesis exists, it should never be used if it can be avoided. Prostration is much more apt to ensue in them, and where the article is persisted in for any length of time, is sure to do harm.

"6. It is perhaps hardly necessary to say that if Tartar Emetic be an article of such danger, the younger the subject to whom it is given, the more likely it is to do harm. In children under a year, I should say, as a general rule, it ought never to be used."—*N. Y. Annalist.*

